# Supplementary material for: Factors associated with a history of treatment interruption among pregnant women living with HIV in Malawi: A cross-sectional study
Source: PLoS One. 2022 Apr 19;17(4):e0267085. doi: 10.1371/journal.pone.0267085 (PMC9017884; doi:10.1371/journal.pone.0267085)
Supplement: S1 File — (PDF) [file pone.0267085.s001.pdf]

S4 MATERNAL ENROLLMENT FORM

Patient Number ☐☐☐☐☐☐–☐☐ Date of Patient Visit ☐☐ ☐☐☐ ☐☐☐☐
D D M M M Y Y Y Y

Form Week ☐☐☐☐

1. What is the participant's date of birth? ☐☐ (dd) ☐☐☐ (MMM) ☐☐ (yy) OR: Age in years: ☐☐
2. What is the participant's current marital status? *Mark only one:*
   - ☐ Single
   - ☐ Married
   - ☐ Living with partner but not married
   - ☐ Separated/married but living separately
   - ☐ Divorced
   - ☐ Widowed
3. During the past 12 months, what was the participant's average **household** monthly income?
   ____________________ Malawi Kwacha OR: ☐ No income
4. During the past 12 months, what was the participant's average **personal** monthly income?
   ____________________ Malawi Kwacha OR: ☐ No income
5. What is the highest level of education the participant has reached? *Mark only one.*
   - ☐ No schooling
   - ☐ Some primary school
   - ☐ Completed primary school
   - ☐ Some secondary school
   - ☐ Completed secondary school
   - ☐ attended college or university (includes vocational or trade school)
   - ☐ Graduate/professional school
6. What's the participant's ethnic group or tribe? *Mark all that apply*:
   - ☐ Chewa
   - ☐ Ngoni
   - ☐ Yao
   - ☐ Tumbuka
   - ☐ Other African tribe, *specify*: _______________ Local language: ________OR: *English*
   - ☐ Other *specify*: _______________ Local language: ________OR: *English*
7. What is the participant's current form of employment? *Mark all that apply.*
   - ☐ Unemployed
   - ☐ Seasonal employment
   - ☐ Farmer
   - ☐ Permanently employed
   - ☐ Student
8. What is the participant's main source of drinking water? *Mark only one*.
   - ☐ Piped water
   - ☐ Unprotected well
   - ☐ Protected

S4 MATERNAL ENROLLMENT FORM

Patient Number ☐☐☐☐☐☐ – ☐☐ Date of Patient Visit ☐☐ ☐☐☐ ☐☐☐☐
D D M M M Y Y Y Y

Form Week ☐☐☐☐

☐ Borehole
☐ Surface water (spring, river/stream, pond/lake, dam)

9. Does the participant have electricity in their home? ☐Y ☐N
10. How long does it take you to get to this clinic from your home? ☐<1hr ☐1-2hrs ☐ >2hrs
11. How far is it from your home to this clinic? ________km OR ☐ Don't know

CURRENT PREGNANCY HISTORY

12. LMP: ☐☐ (dd) ☐☐☐ (MMM) ☐☐ (yy) 12a. EDD: ☐☐ (dd) ☐☐☐ (MMM) ☐☐ (yy)
13. You tested for HIV this week. Was this your first HIV test?
    ☐Y ☐N ☐Don't know ☐No response
    13a. Will this be your first time to start ART because of pregnancy? ☐Y ☐N
    If YES to any label client as cohort A and go to 17
14. Have you ever tested for CD4? ☐Y ☐N
    14a. if YES: result _________ cells/mL 14b. Date of test: ☐☐ (dd) ☐☐☐ (MMM) ☐☐ (yy)
15. Are you already on ART because of this pregnancy? ☐Y ☐N
    If YES label client as cohort B and go to 17
16. Are you already on ART because of reasons other than pregnancy (i.e for your own health)? ☐
    Y ☐N If YES label client as cohort B and go to 17
    16a. Have ever been put on ART for life because you were pregnant previously.
    ☐Y ☐N If YES label client as cohort C

PAST PREGNANCY HISTORY

17. Full term births (>37 weeks) OR: ☐ **None**

**Date of delivery (dd/mmm/yyyy)**	**HIV test**	**PMTCT**
☐☐ (dd) ☐☐☐ (MMM) ☐☐ (yy)	☐Positive<br>☐Negative<br>☐Not done	☐No<br>☐Yes *(specify)*_____________
☐☐ (dd) ☐☐☐ (MMM) ☐☐ (yy)	☐Positive<br>☐Negative<br>☐Not done	☐No<br>☐Yes *(specify)*_____________
☐☐ (dd) ☐☐☐ (MMM) ☐☐ (yy)	☐Positive<br>☐Negative<br>☐Not done	☐No<br>☐Yes *(specify)*_____________

Staff Initials: Filled form_______ Staff Initials: Entered form into Database___
Date __ __/ __ __ __/__ __ __ __ (DD/MMM/YYYY)

S4 MATERNAL ENROLLMENT FORM

Patient Number ☐☐☐☐☐–☐☐ Date of Patient Visit ☐☐ ☐☐☐ ☐☐☐☐
D D MMM Y Y Y Y

Form Week ☐☐☐☐

dd MMM yy<br>☐☐ ☐☐☐ ☐☐	☐Positive<br>☐Negative<br>☐Not done	☐No<br>☐Yes *(specify)*______________

18. Preterm live births (<37 weeks) OR: ☐None

Date of delivery (dd/mmm/yyyy)	HIV test	PMTCT
dd MMM yy<br>☐☐ ☐☐☐ ☐☐	☐Positive<br>☐Negative<br>☐Not done	☐No<br>☐Yes *(specify)*______________
dd MMM yy<br>☐☐ ☐☐☐ ☐☐	☐Positive<br>☐Negative<br>☐Not done	☐No<br>☐Yes *(specify)*______________
dd MMM yy<br>☐☐ ☐☐☐ ☐☐	☐Positive<br>☐Negative<br>☐Not done	☐No<br>☐Yes *(specify)*______________
dd MMM yy<br>☐☐ ☐☐☐ ☐☐	☐Positive<br>☐Negative<br>☐Not done	☐No<br>☐Yes *(specify)*______________

19. **Spontaneous fetal deaths or stillbirths (>20 weeks) OR: ☐None**

Date of delivery (dd/mmm/yyyy)	HIV test	PMTCT
dd MMM yy<br>☐☐ ☐☐☐ ☐☐	☐Positive<br>☐Negative<br>☐Not done	☐No<br>☐Yes *(specify)*______________
dd MMM yy<br>☐☐ ☐☐☐ ☐☐	☐Positive<br>☐Negative<br>☐Not done	☐No<br>☐Yes *(specify)*______________
dd MMM yy<br>☐☐ ☐☐☐ ☐☐	☐Positive<br>☐Negative<br>☐Not done	☐No<br>☐Yes *(specify)*______________
dd MMM yy<br>☐☐ ☐☐☐ ☐☐	☐Positive<br>☐Negative<br>☐Not done	☐No<br>☐Yes *(specify)*______________

20. **Miscarriages (abortions) (<20 weeks) OR: ☐None**

Date of delivery (dd/mmm/yyyy)	HIV test	PMTCT
dd MMM yy<br>☐☐ ☐☐☐ ☐☐	☐Positive<br>☐Negative<br>☐Not done	☐No<br>☐Yes *(specify)*______________

Staff Initials: Filled form________ Staff Initials: Entered form into Database___
Date __ __/ __ __ __/__ __ __ __ (DD/MMM/YYYY)

S4 MATERNAL ENROLLMENT FORM

Patient Number ☐☐☐☐☐–☐☐ Date of Patient Visit ☐☐ ☐☐☐ ☐☐☐☐
D D MMM Y Y Y Y

Form Week ☐☐☐☐

dd MMM yy<br>☐☐ ☐☐☐ ☐☐	☐Positive<br>☐Negative<br>☐Not done	☐No<br>☐Yes *(specify)*_____________
dd MMM yy<br>☐☐ ☐☐☐ ☐☐	☐Positive<br>☐Negative<br>☐Not done	☐No<br>☐Yes *(specify)*_____________
dd MMM yy<br>☐☐ ☐☐☐ ☐☐	☐Positive<br>☐Negative<br>☐Not done	☐No<br>☐Yes *(specify)*_____________

21. **Other pregnancy complications (including fetal anomalies) OR:** ☐**None**

22. How many children does the participant have? ☐☐ Alive ☐☐ Dead
23. Number of children from previous pregnancies, either living or who have died, that meet the CDC criteria for definitive evidence of HIV infection: ☐☐
24. Number of neonatal deaths (≤ 28 days of age): ☐☐

PARTNER INFORMATION:

25. Approximately how old is the sex partner of this pregnancy in years? ☐☐
26. Is this person someone you consider your primary sex partner?
    ☐Y ☐N ☐Don't know ☐No response
27. Do you currently live with this partner?
    ☐Y ☐N ☐Don't know ☐No response
28. What is your partner's current form of employment? *Mark all that apply*:
    - ☐ Unemployed
    - ☐ Seasonal employment
    - ☐ Farmer
    - ☐ Permanently employed
    - ☐ Student
29. What is the highest level of education your partner has reached? *Mark only one.*
    - ☐ No schooling
    - ☐ Some primary school
    - ☐ Completed primary school

S4 MATERNAL ENROLLMENT FORM

Patient Number ☐☐☐☐☐☐–☐☐ Date of Patient Visit ☐☐ ☐☐☐ ☐☐☐☐
D D M M M Y Y Y Y

Form Week ☐☐☐☐

☐ Some secondary school
☐ Completed secondary school
☐ attended college or university (includes vocational or trade school)
☐ Graduate/professional school
☐ Not applicable

30. How long have you been in a relationship or married with this partner? _____________
31. Has this person been tested for HIV in the past?
    ☐Y ☐N ☐Don't know ☐No response
32. What was the result of their last HIV test?
    ☐Y ☐N ☐Don't know ☐No response
33. Have you and this partner ever test for HIV together as a couple?
    ☐Y ☐N ☐Don't know ☐No response
34. Do you intend to tell your partner that you are HIV-positive in the next week?
    ☐Y ☐N ☐Don't know ☐No response
35. If NO to above, reason for not disclosing: partner will:
    ☐Be angry ☐Hurt me ☐ Leave me ☐Share my status with others

PREVIOUS ART HISTORY:

36. Ever taken ART? ☐Y ☐No (SKIP to #38)
37. Indication for ART:

		Regimen	Start Date	Stop Date	Ongoing
Own health	Yes /No	_____	________	________	
PMTCT	Yes/No	_____	________	________	
PEP	Yes/No	_____	________	________	

PATIENT HEALTH QUESTIONS

38. During the past two weeks, how often have you been bothered by each of the following symptoms?

	Not at all	Several days	More than half the days	Nearly every day
1. Feeling down, depressed, or hopeless (*closed spirits*)?	0	1	2	3
2. Little interest or pleasure in doing things; (*not having courage or anxiety*; spirits are low*)?				

Staff Initials: Filled form_______ Staff Initials: Entered form into Database___
Date __ __/ __ __ __/__ __ __ __ (DD/MMM/YYYY)

S4 MATERNAL ENROLLMENT FORM

Patient Number [ ][ ][ ][ ][ ][ ]–[ ][ ] Date of Patient Visit [ ][ ] [ ][ ][ ] [ ][ ][ ][ ]
D D M M M Y Y Y Y

Form Week [ ][ ][ ][ ]

	0	1	2	3
3. Trouble falling or staying asleep (*insomnia, sleeplessness*), or sleeping too much?	0	1	2	3
4. Feeling tired, fatigued or having little energy?	0	1	2	3
5. Poor appetite or overeating?	0	1	2	3
6. Feeling bad about yourself—or that you are a failure or have let yourself or your family down (*feeling ashamed or disgraced*)?	0	1	2	3
7. Trouble concentrating on things, such as:<br><br>participating in meetings or watching television?	0	1	2	3
8. Moving or speaking so slowly that other people could have noticed? Or the opposite—being so fidgety or restless that you have been moving around a lot more than usual (*being a disturbance or not at peace*)?	0	1	2	3
9. Thoughts that you would be better off dead or of hurting yourself in some way? (*feelings of suicide or lost hope*)**	0	1	2	3

Staff Initials: Filled form_______ Staff Initials: Entered form into Database___

Date __ __/ __ __ __/__ __ __ __ (DD/MMM/YYYY)

S4 MATERNAL ENROLLMENT FORM

Patient Number [ ][ ][ ][ ][ ][ ]–[ ][ ] Date of Patient Visit [ ][ ] [ ][ ][ ] [ ][ ][ ][ ]
D D M M M Y Y Y Y

Form Week [ ][ ][ ][ ]

Interviewer: Do these calculations AFTER you are done with the participant. Skip to question 10.	0	× 1 =	× 2 =	× 3 =
Grand total (add totals of columns)				=

[Interviewer: Skip questions 39 and 40 if all responses to questions 1-9 are "0"]

39. How **difficult** have these problems made it for you to do your work, take care of things at home or get along with other people?

[0] Not difficult at all [1] Somewhat difficult [2] Very difficult [3] Extremely difficult

[Interviewer: Skip question 40 if answer to question 39 is "Not difficult at all"]

40. Have they caused you difficulty for two years or more? ☐Y ☐N

Staff Initials: Filled form_______ Staff Initials: Entered form into Database___
Date __ __/ __ __ __/__ __ __ __ (DD/MMM/YYYY)

S4 MATERNAL ENROLMENT FORM (MEF-1-10)

Patient ID ☐☐☐☐-☐☐-☐☐ Date of Patient Visit ☐☐ ☐☐☐ ☐☐☐☐ (D D M M M Y Y Y Y)

EMR # ☐☐☐☐☐☐ Step Number 1 Visit code: 0 0 . 0

1. Kodi otenga nawo mbali anabadwa tsiku liti? 1a. ☐☐ (dd) ☐☐☐ (MMM) ☐☐ (yy) OR, Age (years): 1b. ☐☐
2. *Fill out the below table for __each__ marriage the participant has had. Tick "no" for each marriage that is not applicable (if the participant has never been married, tick "no" in every row).*

Marriage #	i. Applicable?	ii. Outcome/status	iii. Comment
a. Marriage #1	☐$_0$ No ☐$_1$ Yes →	☐$_1$ still married ☐$_2$ divorced ☐$_3$ widowed	
b. Marriage #2	☐$_0$ No ☐$_1$ Yes →	☐$_1$ still married ☐$_2$ divorced ☐$_3$ widowed	
c. Marriage #3	☐$_0$ No ☐$_1$ Yes →	☐$_1$ still married ☐$_2$ divorced ☐$_3$ widowed	
d. Marriage #4	☐$_0$ No ☐$_1$ Yes →	☐$_1$ still married ☐$_2$ divorced ☐$_3$ widowed	

e. Kodi zili bwanji pa moyo wa banja wa otenga nawo mbali? *Mark only one:*

- ☐$_0$ Osakwatiwa (sanakwatiwepo)
- ☐$_1$ Okwatiwa (akukhalira limodzi)
- ☐$_2$ Amakhala ndi chibwenzi koma sanakwatirane
- ☐$_3$ Anasiyana / okwatiwa koma amakhala kosiyana
- ☐$_4$ Banja linatha ndipo sakukhala ndi mwamuna wina aliyense
- ☐$_5$ Mwamuna wawo anamwalira ndipo sakukhala ndi mwamuna wina

3. a. Kodi chimene chimabweretsa ndalama kwambiri ku nyumba kumene mwana wanu azidzakhala ndi chani?
   ☐$_1$ Participant (infant's mother) ☐$_2$ Other family or household member ☐$_3$ Social grant
   ☐$_4$ Pension ☐$_5$ Other (*specify3ai*)___________________ ☐$_9$ Unknown

   b. Kodi ndalama zonse zimene mumazipeza zimakhala zokwanira pa banja lanu?
   ☐$_1$ Yes ☐$_0$ No ☐$_9$ Unknown

4. a. Kodi pamatsala ndalama yapadera pa mwezi imene mungathe kugulira mkaka wa mwana wa m'botolo, mabotolo amwana, mankhwala ophera tidzirombo, kapena ndalama yoyendera kupita kuchipatala inu ndi mwana?(**NOTE: extra money would be money that is available __after__ spending for needs such as food, clothes, school fees**)……. ☐$_1$ Yes ☐$_0$ No ☐$_9$ Unknown
   (No / Unknown) → SKIP to #5

   b. Kodi ndalamazi zikhoza kupezeka chaka chonse? ☐$_1$ Yes ☐$_0$ No ☐$_9$ Unknown

5. Kodi otenga nawo mbali pakadali pano ndalama amazipeza bwanji? *Mark only one.*
   - ☐$_0$ Salipantchito
   - ☐$_1$ Ali pa ntchito yosakhazikika / ganyu
   - ☐$_2$ Ali ndi business yaying'ono
   - ☐$_3$ Mulimi
   - ☐$_4$ Ali pa ntchito yokhazikika
   - ☐$_5$ Ali pa sukulu
6. Kodi otenga nawo mbali adafika nayo pati Sukulu? *Mark only one:*
   - ☐$_0$ Sanaphunzirepo
   - ☐$_1$ Anaphunzirapo pulayimale sukulu
   - ☐$_2$ Anamaliza pulayimale sukulu
   - ☐$_3$ Anaphunzirako sekondale sukulu
   - ☐$_4$ Anamaliza sekondale sukulu
   - ☐$_5$ Anaphunzirako sukulu ya ukachenjede
   - ☐$_6$ Anamaliza sukulu ya ukachenjede
7. Kodi otenga nawo mbali akuchokera ku mtundu wanji? *Mark only one*:
   - ☐$_1$ Chewa
   - ☐$_2$ Ngoni
   - ☐$_3$ Yao
   - ☐$_4$ Tumbuka
   - ☐$_5$ Other *specify*:7i5i ________________

Staff Initials: Filled form_______ Staff Initials: Entered form into Database_______

S4 MATERNAL ENROLMENT FORM (MEF-1-10)

Patient ID ☐☐☐☐-☐☐-☐☐ Date of Patient Visit ☐☐ ☐☐☐ ☐☐☐☐ (D D M M M Y Y Y Y)

EMR # ☐☐☐☐☐☐ Step Number 1 Visit code: 0 0 . 0

8. Kodi otenga nawo mbali amamwa madzi kuchokera kuti? *Mark only one*.
   - ☐ $_0$ Madzi apampopi
   - ☐ $_1$ Chitsime chosatetezedwa
   - ☐ $_2$ Chitsime chotetezedwa
   - ☐ $_3$ Mjigo
   - ☐ $_4$ Madzi a muntsinje (kasupe, stream, nyanja, damu)

9. Kodi otenga nawo mbali ali ndi magetsi kunyumba kwawo? ☐ $_1$ Yes ☐ $_0$ No

10. Kodi zimakutengerani nthawi yayitali bwanji kuchoka kunyumba kubwera kuchipatala kuno?

    ☐ $_0$ < 1hour ☐ $_1$ 1-2hours ☐ $_2$ >2hrs

    10a. Kodi kuchipatala kuno ndikumene mumabwera nthawi zonse mukafuna chithandizo cha zaumoyo?

    ☐ $_1$ Yes → SKIP to #11 ☐ $_0$ No

    10ai. If NO to #10a, Tchulani Chipatala Chimene mumapita nthawi zonse______________________

11. Kodi mumakhala ku dera liti? ____

CURRENT PREGNANCY HISTORY

12. a. LMP: ☐☐ ☐☐☐ ☐☐ (dd MMM yy) 12b. EDD: ☐☐ ☐☐☐ ☐☐ (dd MMM yy)

    12ai ☐ $_9$ Estimated (not exact date)

13. Pa nthawi imene munatenga mimbayi, munafuna kuti mukhale ndi mimba nthawi imeneyo, mmafuna mutadikira musanatenge mimba? kapena simumafuniratu kukhalanso ndi ana?
    - ☐ $_1$ Ndimafuna kutenga mimba nthawi imeneyo
    - ☐ $_2$ Ndimafuna kutenga mimba mtsogolo
    - ☐ $_3$ Sindimafunanso ana ena
    - ☐ $_4$ Sindikudziwa/ sindikukumbukira
    - ☐ $_5$ Palibe yankho

14. Pa nthawi imene munatenga mimba, okondedwa wanu anafuna kuti mukhale ndi mimba nthawi imeneyo, mtsogolo kapena samafuna mkomwe?
    - ☐ $_1$ Okondedwa wanga amafuna nditenge mimba nthawi imeneyo
    - ☐ $_2$ Okondedwa wanga amafuna ndidzatenge mimba mtsogolo
    - ☐ $_3$ Okondedwa wanga samafuna kuti ndikhalenso ndi ana ena
    - ☐ $_4$ Sindikudziwa/ sindikukumbukira
    - ☐ $_5$ Sananyankhe

15. Pa nthawi imene munatenga mimba imeneyi, kodi inuyo kapena okondedwa wanu mumagwiritsa njira ina iriyonse ya kulera? ☐ $_1$ Yes ☐ $_0$ No → SKIP to Question #17

    ☐ $_2$ Don't know ☐ $_3$ No response

16. Ndi njira iti/ziti yolera imene inu kapena okondedwa wanu mumagwiritsa ntchito? (*check "Yes" for ALL methods that are mentioned as being used; check "No" if method not used)*

a. Abstinence	☐ $_1$ Yes ☐ $_0$ No
b. Natural family planning (timed intercourse, etc.)	☐ $_1$ Yes ☐ $_0$ No
c. Breastfeeding	☐ $_1$ Yes ☐ $_0$ No
d. Withdrawal (pulling out)	☐ $_1$ Yes ☐ $_0$ No
e. Male/Female condoms	☐ $_1$ Yes ☐ $_0$ No
f. Birth control pill<br>fi. If YES, date of most recent pill taken (dd/mmm/yy):	☐ $_1$ Yes ☐ $_0$ No<br>__ __/ __ __ __/ __ __
g. Birth control injection<br>gi. If YES, date of most recent injection (dd/mmm/yy):	☐ $_1$ Yes ☐ $_0$ No<br>__ __/ __ __ __/ __ __
h. Birth control implant	☐ $_1$ Yes ☐ $_0$ No

Staff Initials: Filled form_______ Staff Initials: Entered form into Database_______

S4 MATERNAL ENROLMENT FORM (MEF-1-10)

Patient ID ☐☐☐☐-☐☐-☐☐ Date of Patient Visit ☐☐ ☐☐☐ ☐☐☐☐ (D D M M M Y Y Y Y)

EMR # ☐☐☐☐☐☐ Step Number 1 Visit code: 0 0 . 0

hi. If YES, *specify type of implant:*<br>hii. Date of insertion (dd/mmm/yy):	☐ 1 Jadelle ☐ 2 Other<br>__ __/__ __ __/__ __
i. Intrauterine device (IUD)<br>ii. If YES, date of insertion (dd/mmm/yy):	☐ 1 Yes ☐ 0 No<br>__ __/__ __ __/__ __
j. Female sterilization (tubal ligation)	☐ 1 Yes ☐ 0 No
k. Male sterilization (vasectomy)	☐ 1 Yes ☐ 0 No
l. Other (***specify***):	☐ 1 Yes ☐ 0 No
m. Don't know	☐ 1 Yes ☐ 0 No

17. What is the participant's current WHO stage? **NOTE: read from Mastercard** ☐
18. Do you (does the participant) have any pre-existing medical conditions? ☐ 1 Yes ☐ 0 No

******Complete Pre-existing Medical Conditions and WHO Staging form******

19. Pregnancy measurements:

19a. Fundal height: ☐☐ cm	☐ 98 N/A (< 24 weeks)		☐ 99 Information unavailable
19b. Fetal heart tones:	☐ 1 Present<br>☐ 3 N/A (<24 weeks)	☐ 0 Absent	☐ 2 Abnormal (*specify*) 19bi:____________<br>☐ 99 Information unavailable
19c. Ultrasound dating:	☐ 0 Not done	☐ 1 Done →	19ci. ☐☐ weeks & ☐ days

PAST PREGNANCY HISTORY

20. Complete table below for each pregnancy. **If NO PRIOR PREGNANCY, CHECK** ☐ **0 None →SKIP to #24**

NOTE: **"Term" means live births born at ≥ 37 weeks gestation.**
"Preterm" means live births born at <37 weeks gestation
"Stillbirth" means stillbirths or spontaneous fetal deaths at ≥20 weeks gestation
"Abortion" means miscarriage or abortion at <20 weeks gestation

**i. Date of delivery or miscarriage**	**ii. Delivery Type**	**iii. Maternal HIV test**	**iv. PMTCT**
1. Pregnancy #1<br>*dd* ☐☐ *MMM* ☐☐☐ *yy* ☐☐	☐ 0 Term (≥37weeks)<br>☐ 1 Preterm (<37weeks)<br>☐ 2 Stillbirth (≥20weeks)<br>☐ 3 Abortion (<20weeks)	☐ 0 Negative<br>☐ 1 Positive<br>☐ 2 Indeterminate<br>☐ 3 Don't know<br>☐ 9 Not done	☐ 9 N/A (HIV-negative during this pregnancy)<br>☐ 0 No<br>☐ 1 Yes *(specify)v.:*____________<br>______________________
2. Pregnancy #2<br>*dd* ☐☐ *MMM* ☐☐☐ *yy* ☐☐	☐ 0 Term (≥37weeks)<br>☐ 1 Preterm (<37weeks)<br>☐ 2 Stillbirth (≥20weeks)<br>☐ 3 Abortion (<20weeks)	☐ 0 Negative<br>☐ 1 Positive<br>☐ 2 Indeterminate<br>☐ 3 Don't know<br>☐ 9 Not done	☐ 9 N/A (HIV-negative during this pregnancy)<br>☐ 0 No<br>☐ 1 Yes *(specify)v.:*____________<br>______________________
3. Pregnancy #3<br>*dd* ☐☐ *MMM* ☐☐☐ *yy* ☐☐	☐ 0 Term (≥37weeks)<br>☐ 1 Preterm (<37weeks)<br>☐ 2 Stillbirth (≥20weeks)<br>☐ 3 Abortion (<20weeks)	☐ 0 Negative<br>☐ 1 Positive<br>☐ 2 Indeterminate<br>☐ 3 Don't know<br>☐ 9 Not done	☐ 9 N/A (HIV-negative during this pregnancy)<br>☐ 0 No<br>☐ 1 Yes *(specify)v.:*____________<br>______________________
4. Pregnancy #4<br>*dd* ☐☐ *MMM* ☐☐☐ *yy* ☐☐	☐ 0 Term (≥37weeks)<br>☐ 1 Preterm (<37weeks)<br>☐ 2 Stillbirth (≥20weeks)<br>☐ 3 Abortion (<20weeks)	☐ 0 Negative<br>☐ 1 Positive<br>☐ 2 Indeterminate<br>☐ 3 Don't know<br>☐ 9 Not done	☐ 9 N/A (HIV-negative during this pregnancy)<br>☐ 0 No<br>☐ 1 Yes *(specify)v.:*____________<br>______________________
5. Pregnancy #5<br>*dd* ☐☐ *MMM* ☐☐☐ *yy* ☐☐	☐ 0 Term (≥37weeks)<br>☐ 1 Preterm (<37weeks)<br>☐ 2 Stillbirth (≥20weeks)<br>☐ 3 Abortion (<20weeks)	☐ 0 Negative<br>☐ 1 Positive<br>☐ 2 Indeterminate<br>☐ 3 Don't know	☐ 9 N/A (HIV-negative during this pregnancy)<br>☐ 0 No<br>☐ 1 Yes *(specify)v.:*____________

Staff Initials: Filled form_______ Staff Initials: Entered form into Database_______

S4 MATERNAL ENROLMENT FORM (MEF-1-10)

Patient ID ☐☐☐☐-☐☐-☐☐   Date of Patient Visit ☐☐ ☐☐☐ ☐☐☐☐ (D D M M M Y Y Y Y)

EMR # ☐☐☐☐☐☐   Step Number [1]   Visit code: [0][0].[0]

i. Date of delivery or miscarriage	ii. Delivery Type	iii. Maternal HIV test	iv. PMTCT
		☐ 9 Not done	______________________
6. Pregnancy #6<br>dd ☐☐ MMM ☐☐☐ yy ☐☐	☐ 0 Term (≥37weeks)<br>☐ 1 Preterm (<37weeks)<br>☐ 2 Stillbirth (≥20weeks)<br>☐ 3 Abortion (<20weeks)	☐ 0 Negative<br>☐ 1 Positive<br>☐ 2 Indeterminate<br>☐ 3 Don't know<br>☐ 9 Not done	☐ 9 N/A (HIV-negative during this pregnancy)<br>☐ 0 No<br>☐ 1 Yes *(specify)v.:*_____________<br>______________________

21. **Other pregnancy complications (including fetal anomalies) OR: ☐ 0 None**

	i. PREGNANCY NUMBER (1st, 2nd, etc.)	ii. COMPLICATION/ANOMALY	iii. CODE (refer to appendix 1)
a.			
b.			
c.			

22. a. Kodi otenga nawo mbali ali ndi ana angati? ……# Amoyo ☐☐
    22b. Ana omwalira alipo angati (kuphatikiza kupita pa dera) ☐☐ (if no child died, SKIP to #23)
    22c. Pa ana omwalira, ndi angati munapititsa padera (≤ 28 days of age)?: …….. ☐☐
23. Number of children from previous pregnancies, either living or who have died, that meet Malawi HIV 2014 guidelines for definitive evidence of HIV infection: ☐☐

PARTNER INFORMATION: *note: #25-37a* ***answer for current partner*** *if man is different from sex partner of pregnancy*

24. Kodi muli pa ubwenzi ndi mwamuna amene ndi mwini wake wa mimbayi?
    ☐ 1 Yes ☐ 0 No ☐ 2 Don't know ☐ 3 No response
25. Ndi nthawi yaitali bwanii imene mwakhala paubwenzi kapena pabanja ndi mwamuna ameneyu?
    (fill in BOTH yr & mo) ☐☐ a(years) & ☐☐ b(months) ☐ 99 No response
26. Kodi mwamuna ameneyu ndi amene ali ogonana naye okhazikika?
    ☐ 1 Yes ☐ 0 No ☐ 2 Don't know ☐ 3 No response
27. Kodi pakadali pano mumakhala limodzi ndi mwamuna ameneyu?
    ☐ 1 Yes ☐ 0 No ☐ 2 Don't know ☐ 3 No response
28. Kodi mwamuna ameneyi ali ndi zaka pafupifupi zingati? ☐☐
29. Kodi mwamuna ameneyi amapeza ndalama munjira zanji? *Mark only one*:
    - ☐ 0 Sali pa ntchito
    - ☐ 1 Ali pa ntchito yosakhazikika
    - ☐ 2 Small vendor
    - ☐ 3 Mulimi
    - ☐ 4 Ali pa ntchito yokhazikika
    - ☐ 5 Ali pa sukulu
30. Kodi mwamuna ameneyi sukulu adafika nayo pati? *Mark only one.*
    - ☐ 0 Sanaphunzirepo
    - ☐ 1 Anaphunzirapo ku pulayimale sukulu
    - ☐ 2 Anamaliza pulayimale sukulu
    - ☐ 3 Anaphunzirapo ku sekondale sukulu
    - ☐ 4 Anamaliza sekondale sukulu
    - ☐ 5 Anaphunzirapo sukulu ya ukachenjede
    - ☐ 6 Anamaliza sukulu ya ukachenjede

Staff Initials: Filled form_______   Staff Initials: Entered form into Database_______

S4 MATERNAL ENROLMENT FORM (MEF-1-10)

Patient ID ☐☐☐☐-☐☐-☐☐ Date of Patient Visit ☐☐ ☐☐☐ ☐☐☐☐ (D D M M M Y Y Y Y)

EMR # ☐☐☐☐☐☐ Step Number 1 Visit code: 0 0 . 0

31. Kodi mwamuna ameneyu anayezetsapo HIV mmbuyomu?
    ☐ 1 Yes ☐ 0 No ☐ 2 Don't know ☐ 3 No response → SKIP TO QUESTION #33

31a. Ngati Eya, tsiku lake lotsiriza kuyezetsa HIV linali liti?: (MMM/YYYY) __ __ __/__ __ __ __

32. Kodi zotsatira zomaliza za HIV zinali zotani?
    ☐ 1 Positive ☐ 0 Negative ☐ 2 Indeterminate ☐ 3 Don't know ☐ 4 No response

33. Kodi inu ndi okondedwa wanuyu munayamba mwayezetsa HIV limodzi?
    ☐ 1 Yes → 33a. Date of your most recent partner HIV test (MMM/YYYY) __ __ __/ __ __ __ __
    ☐ 0 No ☐ 2 Don't know ☐ 3 No response → SKIP TO QUESTION #34

33b. Ngati Eya, zotsatitira zoyezetsa zinali zotani?
- ☐ 0 Concordant negative
- ☐ 1 Concordant positive → SKIP TO QUESTION #36
- ☐ 2 Discordant
- ☐ 3 Don't know
- ☐ 4 No response

34. Kodi mukuyembekeza kumuuza okondedwa wanu kuti muli ndi HIV??
    ☐ -0 No → continue to #35
    ☐ 1 Yes ☐ 2 I've already told him ☐ 3 Don't know ☐ 4 No response → SKIP to #36

35. Zifukwa zosaululira kuti ndili ndi HIV: kuopa kuti okondedwa wanga akhoza/akanakhoza;
    35a. Kundikwiyira………………………………………… ☐ 1 Yes ☐ 0 No ☐ 2 No response
    35b. Kundivulaza…………………………………………. ☐ 1 Yes ☐ 0 No ☐ 2 No response
    35c. Kundisiya…………………………………………….. ☐ 1 Yes ☐ 0 No ☐ 2 No response
    35d. Kufotokozera anthu ena kuti ndili ndi HIV….. ☐ 1 Yes ☐ 0 No ☐ 2 No response
    35e. Zifukwa zina *(fotokozani)*:____

36. Kodi amuna anuwa anayamba akuopsezani?
    ☐ 1 Yes ☐ 0 No ☐ 2 No response → SKIP to #37

36a. Ngati Eya, wakuopsezaniko miyezi itatu yapitayi?
    ☐ 1 Yes ☐ 0 No ☐ 2 No response

37. Kodi munthu ameneyu anayamba wakuvulazani?
    ☐ 1 Yes ☐ 0 No ☐ 2 No response → SKIP to #38

37a. Ngati Eya, wakuvulazaniko miyezi itatu yapitayi?
    ☐ 1 Yes ☐ 0 No ☐ 2 No response

S4 MATERNAL ENROLMENT FORM (MEF-1-10)

Patient ID ☐☐☐☐-☐☐-☐☐ Date of Patient Visit ☐☐ ☐☐☐ ☐☐☐☐ D D M M M Y Y Y Y

EMR # ☐☐☐☐☐☐ Step Number 1 Visit code: 0 0 . 0

PATIENT HEALTH QUESTIONS (PHQ-9)

[Read to participant: Tsopano ndikufunsani mafuso okhuzana ndi m'mene mwakhala mukumvera masabata awiri apitawo. Chonde ndiuzeni ngati mwakhala mukumva motere: Olo M'pang'ono pomwe, Matsiku angapoanu ndi limodzi, pafuifupi theka la masiku onse, kapena pafupifupi tsiku lirilonse.***]***

38. ***Mumasabata awiri apitawa***, ndikangati munavutika ndi zizindikiro zotsatirazi?

	Mpangonong'ono-pomwe (0)	Masiku angapoanu ndi limodzi (1-7 days)	Pafupifupi theka la masiku onse (8-11 days)	Pafupifupi tsiku lirilonse (12-14 days)
1. Kumva kukhumudwa, kupanda chiyembekezo?	☐ 0	☐ 1	☐ 2	☐ 3
2. Chikhumbo chochepa kapena kusasangalatsidwa mkuchita zinthu; (osalimba mtima kapena kukhala ndi nkhawa)?	☐ 0	☐ 1	☐ 2	☐ 3
3. Kuvutika kupeza tulo kapena kugonetsa (kusowa tulo, kugonetsa)?	☐ 0	☐ 1	☐ 2	☐ 3
4. Kufooka kapena kuchepekedwa mphamvu?	☐ 0	☐ 1	☐ 2	☐ 3
5. Kusowa chilakolako cha chakudya kapena kudya kwambiri?	☐ 0	☐ 1	☐ 2	☐ 3
6. Kusamva bwino za iwe mwini —kapena kuziona olephera, okanika kuziyang'anira wekha kapena a pa banja ako (kapena kuzimvera manyazi/chitonzo wekha)?	☐ 0	☐ 1	☐ 2	☐ 3
7. Kukanika kukhazikika pa chinthu, monga; [ingati wamwamuna] kukanika kuwerenga nyuzi pepala kapena kuonera kanema [ngati wamkazi] kukanika kutenganawo mbali munsonkhano kapena kuonera kanema?	☐ 0	☐ 1	☐ 2	☐ 3
8. Kuyenda kapena kuyankhula mochedwa zoti mpakana anthu ena kudabwa kapena kusakhazikika nkumangoyenda yenda moposera muyeso (kukhala osokoneza kapena kusowa mtendere)?	☐ 0	☐ 1	☐ 2	☐ 3
9. Malingaliro akuti kuli bwino kungofa kudzida wekha mwa njira ina yake? (*kufuna kuzipha kapena kutaya chiyembekezo)*****Refer to S4 clinician for any score other than 0***	☐ 0	☐ 1	☐ 2	☐ 3
TOTAL (per column; sum of columns should equal 9)				
Interviewer: Do these calculations AFTER you are done with the participant. Skip to question 39.	x 0= 0	x 1=	x 2=	x 3=
GRAND TOTAL (add all columns; min=0, max=27)				=

[Interviewer: SKIP questions 39 and 40 if all responses to questions 38.1-9 are "0"]

39. Kodi zinali **zovuta** bwanji kwa inu kukwaniritsa kugwira ntchito yanu, kusamalira zinthu pakhomo panu kapena kukhala ndi anthuzi chifukwa cha zovuta zimenezi?

☐ 0 Zosavuta nkomwe ☐ 1 Zovutirapo ☐ 2 Zovuta kwambiri ☐ 3 Zovutitsitsa

→ SKIP to #41.1 (EPDS)

40. Zinabweretsa zovuta kwa zaka ziwiri kapena kuposera apo? ☐ 1 Inde ☐ 0 Ayi

Staff Initials: Filled form_______ Staff Initials: Entered form into Database_______

S4 MATERNAL ENROLMENT FORM (MEF-1-10)

Patient ID ☐☐☐☐-☐☐-☐☐ Date of Patient Visit ☐☐ ☐☐☐ ☐☐☐☐ (D D M M M Y Y Y Y)

EMR # ☐☐☐☐☐☐ Step Number 1 Visit code: 0 0 . 0

Edinburgh Postnatal Depression Scale (EPDS): questions 41.1-41.10 ***[Interviewer: read this to the participant:***
Tsopano ndikufunsani mafunso a mmene mwakhala mukuganizila ndikumvera masiku asanu ndi awiri apitawa. Mafunso awiri oyambilira tigwilitsa ntchito mbali imodzi ya kadi. Chithunzi chilichonse chikuimila limodzi mwa mayankho anayi. Ndidziloza zithunzi ndikamawelenga mayankho a funso lililonse. Musankhe yankho logwilizana ndi m'mene mwakhala mukumvela masiku asanu ndi awiri apitawa.*"]*

41.1	***Masiku asanu ndi awiri apitawa:***<br>...kodi mwakhala mukutha kuseka komanso kuona kusangalatsa kwa zinthu?	☐ $_0$ Monga m'mene mumathera nthawi zonse<br>☐ $_1$ Osati bwino kwambiri<br>☐ $_2$ Panopa osati kwambiri<br>☐ $_3$ Olo mpang'ono komwe
41.2	***Masiku asanu ndi awiri apitawa:***<br>**…** kodi mwakhala mukudikira ndi nsangala mu zinthu zozachitika mtsogolo?	☐ $_0$ Monga m'mene mumathera nthawi zonse<br>☐ $_1$ Osati bwino kwambiri<br>☐ $_2$ Panopa osati kwambiri<br>☐ $_3$ Olo mpang'ono komwe
41.3	***Masiku asanu ndi awiri apitawa*:**<br>**…** kodi mumazida nokha mosafunikila pamene zinthu sizinayendebwino?	☐ $_3$ Nthawi zambiri<br>☐ $_2$ Kawirikawiri<br>☐ $_1$ Mwakamodzikamodzi<br>☐ $_0$ Sizinachitikepo
41.4	***Masiku asanu ndi awiri apitawa*:**<br>**…** kodi mumakhumudwa kapena kudela nkhawa popanda chifukwa chenicheni?	☐ $_0$ Olo mpang'ono pomwe<br>☐ $_1$ Sizimachitika<br>☐ $_2$ Nthawi zina<br>☐ $_3$ Kwambiri
41.5	***Masiku asanu ndi awiri apitawa*:**<br>**…** kodi mumachita mantha kapena kusowa mtendere popanda chifukwa chenicheni**?**	☐ $_3$ Kwambiri<br>☐ $_2$ Nthawi zina<br>☐ $_1$ Osati kwambiri<br>☐ $_0$ Ngakhale pang'ono
41.6	***Masiku asanu ndi awiri apitawa:***<br>**…** kodi mwakhala mukuganiza kapena kumva ngati munalindi zinthu zambiri zoyenela kuchita koma simumakwanisa kuchita?	☐ $_3$ Nthawi zambiri mwakhala mukulepheratu<br>☐ $_2$ Nthawi zina mwakhala mukulepheratu<br>☐ $_1$ Nthawi zambiri mwakhala mukutha<br>☐ $_0$ Mwakhala mukutha ngati m'mene mumapangira nthawi
41.7	***Masiku asanu ndi awiri apitawa:***<br>**…** kodi mwakhala osasangalala moti mwakhala mukulephera kugona**?**	☐ $_3$ Nthawi zambiri<br>☐ $_2$ Kawirikawiri<br>☐ $_1$ Osati kawirikawiri<br>☐ $_0$ Mpang'ono pomwe
41.8	***Masiku asanu ndi awiri apitawa:***<br>**…** kodi munali wokhumudwa kapena kusowa mtendere wa mumtima**?**	☐ $_3$ Nthawi zambiri<br>☐ $_2$ Kawirikawiri<br>☐ $_1$ Osati kawirikawiri<br>☐ $_0$ Mpang'ono pomwe
41.9	***Masiku asanu ndi awiri apitawa:***<br>**…** kodi mwakhala osasangalala moti mwakhala mukulira?	☐ $_3$ Nthawi zambiri<br>☐ $_2$ Kawirikawiri<br>☐ $_1$ Mwakamodzikamodzi<br>☐ $_0$ Sizinachitikepo
41.10	***Masiku asanu ndi awiri apitawa:***<br>**…** kodi munakhalapo ndi maganizo ofuna kuzipweteka**?**	☐ $_3$ Nthawi zambiri<br>☐ $_2$ Kawirikawiri<br>☐ $_1$ Mwakamodzikamodzi<br>☐ $_0$ Sizinachitikepo
**TOTAL EPDS SCORE (tally AFTER finish form- see instructions after #45)**		☐☐ (min=0, max=30)

Staff Initials: Filled form_______ Staff Initials: Entered form into Database_______

S4 MATERNAL ENROLMENT FORM (MEF-1-10)

Patient ID ☐☐☐☐ - ☐☐ - ☐☐ Date of Patient Visit ☐☐ ☐☐☐ ☐☐☐☐
D D M M M Y Y Y Y

EMR # ☐☐☐☐☐☐ Step Number 1 Visit code: 0 0 . 0

42. Ku banja kwanu alipo amene ali ndi vuto la kukhumudwa kapena kuda nkhawa?
☐ 1 Yes ☐ 0 No ☐ 2 Don't know
43. Kodi mwakhalapo okhumudwa kapena kuda nkhawa mmbuyomu?
☐ 1 Yes ☐ 0 No ☐ 2 Don't know
44. Kodi munayamba mwayeserako kudzipha nokha?
☐ 1 Yes ☐ 0 No ☐ 2 Don't know

45. Kodi kumvetsetsa kwanu kwa program ya **Option B+** kunali/nkotani? *(allow participant to answer freely before asking items 45b-f; record response here)*: ____________________

____

____

____

Kodi mukudziwa za Option B+ mmadera awa…: *(ask about each item)*

45b. kuti mankhwala a Option B+ ndi akumwa moyo onse:	☐ 1 Yes	☐ 0 No
45c. Kuti mankhwala ndiothandiza kuti mwana asatengere HIV basi:	☐ 1 Yes	☐ 0 No
45d. Kuti mankhwala ndi othandiza thanzi la amayi:	☐ 1 Yes	☐ 0 No
45e. Kuti mankhwala ndi othandiza thanzi la mwana:	☐ 1 Yes	☐ 0 No

45f. Zina (*fotokozani*): ____

EPDS SCORING INSTRUCTIONS:

- Maximum score: 30
- Possible depression: score of 10 or greater
- *Always look at item 41.10 (suicidal thoughts)- refer to S4 clinician*
- **All questions are scored as indicated by the number next to the statement's corresponding box**:
  - Questions 41.1, 2, & 4 are scored 0, 1, 2, or 3 with top box scored as 0 and bottom box scored as 3
  - Questions 41.3, 5-10 are reverse scored, with the top box scored as 3 and the bottom box scored as 0

[END OF FORM]

S4 DEFAULTER QUESTIONNAIRE (DQ-1)

Patient ID ☐☐☐☐-☐☐-☐☐ Date of Patient Visit ☐☐ ☐☐☐ ☐☐☐☐ (D D M M M Y Y Y Y)

EMR # ☐☐☐☐☐☐ Step Number 1 Visit code: 0 0 . 0

PREVIOUS HIV TESTING & PMTCT HISTORY: [Read to participant: all questions relate to the pregnancy in which you most recently defaulted from Option B+]

1a. Did you (the participant) default during **THIS** current pregnancy? ☐0 No ☐1 Yes → SKIP to #11

#	Item	Date (dd/mmm/yy)	i. Estimated date?
1.	First HIV test on enrolling in Option B+ program	dd ☐☐ MMM ☐☐☐ yy ☐☐	☐9 Estimated ☐1 Actual date
2.	Start date of Option B+ in pregnancy in which most recently defaulted	dd ☐☐ MMM ☐☐☐ yy ☐☐	☐9 Estimated ☐1 Actual date
3.	Stop date of Option B+ in pregnancy in which most recently defaulted	dd ☐☐ MMM ☐☐☐ yy ☐☐	☐9 Estimated ☐1 Actual date

4. Tested with partner (of Option B+ pregnancy in which most recently defaulted)……………………… ☐1 Yes ☐0 No
5. Disclosed test results to partner (of Option B+ pregnancy in which most recently defaulted):….. ☐1 Yes ☐0 No
6. Disclosed test result to any other family members (in pregnancy in which most recently defaulted): ☐1 Yes ☐0 No
7. Infant from most recently defaulted Option B+ pregnancy: HIV & Vital status

Child #	1. HIV Status	2. Vital Status
a. 1 (singleton or 1st of multiple births)	☐0 HIV negative<br>☐1 HIV positive<br>☐2 HIV status unknown	☐0 Alive<br>☐1 Neonatal death (<28 days old)<br>☐2 Infant death (<1 year old)
b. 2 (2nd of multiple births)<br>☐1 Applicable ----------→<br>☐0 N/A → SKIP to #8	☐0 HIV negative<br>☐1 HIV positive<br>☐2 HIV status unknown	☐0 Alive<br>☐1 Neonatal death (<28 days old)<br>☐2 Infant death (<1 year old)

8. Marital status at pregnancy in which most recently defaulted in Option B+:
   - ☐0 Single (never married)
   - ☐1 Married (living together)
   - ☐2 Living with partner but not married
   - ☐3 Separated/married but living separately
   - ☐4 Divorced (and not living with any new partner)
   - ☐5 Widowed (and not living with any new partner)
9. Is the partner of this pregnancy the same as the Option B+ pregnancy in which you most recently defaulted? ☐1 Yes ☐0 No
10. Did you change location (where you live) after this most recent Option B+ pregnancy? ☐1 Yes ☐0 No
11. a. What are your (the participant's) reason(s) for **defaulting** from the Option B+ program? (*allow participant to answer freely before asking items 11b-v; record response here*):

    ____

*[**Read to participant**: I will ask you about different reasons why you defaulted. Please tell me whether the reason contributed "a lot", "a little" or "none" to why you stopped Option B+ ART.]*

How much did each of these items contribute to why you defaulted? *ask about **each** item b-v*

11b. Simply did not want to take drugs anymore:	☐2 A lot	☐1 A little	☐0 None
11c. Religious reasons believed was cured after prayers	☐2 A lot	☐1 A little	☐0 None
11d. Traditional/spiritual beliefs	☐2 A lot	☐1 A little	☐0 None
11e. Fear of side effects	☐2 A lot	☐1 A little	☐0 None
11f. No longer had support of partner (separated/divorced)	☐2 A lot	☐1 A little	☐0 None
11g. Was not feeling ill	☐2 A lot	☐1 A little	☐0 None

Staff Initials: Filled form_______ Staff initials: entered form into database: ____

S4 DEFAULTER QUESTIONNAIRE (DQ-1)

Patient ID ☐☐☐☐-☐☐-☐☐ Date of Patient Visit ☐☐ ☐☐☐ ☐☐☐☐ (D D M M M Y Y Y Y)

EMR # ☐☐☐☐☐☐ Step Number 1 Visit code: 0 0 . 0

How much did each of these items contribute to why you defaulted? ***ask about <u>each</u> item b-v***	
11h. Believed in alternate PMTCT	☐2 A lot ☐1 A little ☐0 None
11hi: If "a lot" or "a little", *specify*:	☐1 alternate medicine<br>☐2 herbal medicine
11i. Stigma	☐2 A lot ☐1 A little ☐0 None
11ii: if "a lot" or "a little", *specify (check all that apply)*:	☐1 Immediate family stigma<br>☐2 Extended family stigma<br>☐3 Community stigma<br>☐4 Other *(specify)*:11ii4i________________
11j. Delivered baby and so did not see the need for ART	☐2 A lot ☐1 A little ☐0 None
11k. Stopped breastfeeding so did not see the need for ART	☐2 A lot ☐1 A little ☐0 None
11l. Partner/husband influenced to stop	☐2 A lot ☐1 A little ☐0 None
11m. Influential family member influenced to stop	☐2 A lot ☐1 A little ☐0 None
11n. Influential community member influenced to stop	☐2 A lot ☐1 A little ☐0 None
11ni. If "a lot" or "a little", *specify* which community member:	
11o. No time to go for ART care	☐2 A lot ☐1 A little ☐0 None
11oi. If "a lot" or "a little", *specify*	☐1 Farming<br>☐2 Small business holder<br>☐3 Employer not allowing<br>☐4 Travel<br>☐5 Other *(specify)*:*11oi5i* ______________
11p. ART clinic too far	☐2 A lot ☐1 A little ☐0 None
11q. Poor service at ART clinic	☐2 A lot ☐1 A little ☐0 None
11qi. If "a lot" or "a little", *specify (check all that apply)*:	☐1 Lack of ART<br>☐2 Erratic ART supply<br>☐3 Long waiting hours<br>☐4 No staff at clinic<br>☐5 Attitude of staff at PMTCT clinic<br>☐6 Other (*specify*):11qi6i:______________
11r. Wanted private ART clinic	☐2 A lot ☐1 A little ☐0 None
11s. Side effects of ART	☐2 A lot ☐1 A little ☐0 None
11si. If "a lot" or "a little", *specify (check all that apply):*	☐1 CNS symptoms<br>☐2 GI symptoms<br>☐3 Rash<br>☐4 Jaundice<br>☐5 Renal problems<br>☐6 Other (*specify*): 11si6i:________________
11t. Believed her CD4 count was high enough	☐2 A lot ☐1 A little ☐0 None
11u. Infant outcome	☐2 A lot ☐1 A little ☐0 None
11ui. If "a lot" or "a little", *specify*	
11v. Other	☐2 A lot ☐1 A little ☐0 None
11vi. If "a lot" or "a little", *specify*	

Staff Initials: Filled form_______ Staff initials: entered form into database: ____

S4 DEFAULTER QUESTIONNAIRE (DQ-1)

Patient ID ☐☐☐☐ - ☐☐ - ☐☐ Date of Patient Visit ☐☐ ☐☐☐ ☐☐☐☐
D D M M M Y Y Y Y

EMR # ☐☐☐☐☐☐ Step Number 1 Visit code: 0 0 . 0

12. a. What are your (the participant's) reason(s) for **re-entry** into the Option B+ program? (*allow participant to answer freely before asking items 12b-k; record response here)*:

____

____

[***Read to participant****: I will ask you about different reasons why you have come back for Option B+ ART. Please tell me whether the reason contributed "a lot", "a little" or "none" to why you returned for Option B+ ART.]*

How much does each of these items contribute to your (the participant's) **re-entry** into Option B+?
ask about ***each*** *item b-k*

12b. Subsequent antenatal education program	☐ $_2$ A lot ☐ $_1$ A little ☐ $_0$ None
12c. Because of current pregnancy (came only for PMTCT in this pregnancy)	☐ $_2$ A lot ☐ $_1$ A little ☐ $_0$ None
12d. Has been re-educated on Option B+ but not in the current ANC:	☐ $_2$ A lot ☐ $_1$ A little ☐ $_0$ None
12di. If "a lot" or "a little", *specify*	☐ $_1$ Other ART clinic<br>☐ $_2$ Inpatient<br>☐ $_3$ OPD<br>☐ $_4$ Other community health programmes
12e. Maternal Illness:	☐ $_2$ A lot ☐ $_1$ A little ☐ $_0$ None
12ei: if "a lot" or "a little", *specify*	Illness:
12f. Not ill now (initially was too sick to come to clinic):	☐ $_2$ A lot ☐ $_1$ A little ☐ $_0$ None
12g. Previous infant status:	☐ $_2$ A lot ☐ $_1$ A little ☐ $_0$ None
12gi. & gii. If "a lot" or "a little", *specify* infant HIV and VITAL status:	HIV STATUS: 12gi.<br>☐ $_0$ HIV-uninfected<br>☐ $_1$ HIV-infected<br>☐ $_2$ HIV-status unknown<br><br>VITAL STATUS: 12gii.<br>☐ $_0$ Alive<br>☐ $_1$ Neonatal death<br>☐ $_2$ Infant death
12h. Low CD4 count	☐ $_2$ A lot ☐ $_1$ A little ☐ $_0$ None
12i. Improved PMTCT services	☐ $_2$ A lot ☐ $_1$ A little ☐ $_0$ None
12ii. If "a lot" or "a little", specify *(check all that apply)*:	☐ $_1$ ART supply has improved<br>☐ $_2$ Staff attitude has improved<br>☐ $_3$ Shorter waiting hours<br>☐ $_4$ Better staffing levels at clinic<br>☐ $_5$ Better access to ART clinic *(specify)*: 12ii5: ______________________<br>☐ $_6$ Other *(specify)*: 12ii6: ____________________
12j. Resolution of stigma	☐ $_2$ A lot ☐ $_1$ A little ☐ $_0$ None
12ji. If "a lot" or "a little", specify:	
12k. Other reason	☐ $_2$ A lot ☐ $_1$ A little ☐ $_0$ None
12ki. If "a lot" or "a little", specify:	

[END OF FORM]

Staff Initials: Filled form_______ Staff initials: entered form into database: ____

S4 DEFAULTER QUESTIONNAIRE (DQ-1)

Patient ID ☐☐☐☐-☐☐-☐☐ Date of Patient Visit ☐☐ ☐☐☐ ☐☐☐☐ (D D M M M Y Y Y Y)

Step Number ☐ Visit code: ☐☐.☐

HIV TESTING & PMTCT HISTORY:

A. Ndi chifukwa chiyani inu (otenga mbali) munayamba kumwa ma ARV?

☐ 1 Option B+ (muli ndi mimba kapena mukuyamwitsa) ☐ 2 Thanzi lanu

B. Kodi inu (otenga mbali) munasiya kumwa ma ARV pa mimba iyi?

☐ 0 Ayi ☐ 1 Inde → SKIP to #11 ☐ 9 N/A (started for own health)

#	Item	a. Date (dd/mmm/yy)	i. Estimated date?
1.	Tsiku loyamba kuyezetsa magazi musanayambe kumwa ma ARV:	__ __ / __ __ __ / __ __	☐ 9 Estimated ☐ 1 Actual
2.	Tsiku lotsiriza kumwa ma ARV: *(osati mankhwala amene akumwa pano ngati otenga mbali ayambiranso kumwa ma ARV)*	__ __ / __ __ __ / __ __	☐ 9 Estimated ☐ 1 Actual
3.	Tsiku lomwe mwasiya kumwa ma ARV:	__ __ / __ __ __ / __ __	☐ 9 Estimated ☐ 1 Actual

Pa mankhwala amene mumamwa ndipo mwasiya, kodi muna:

4. Yezetsa limodzi ndi okendedwa anu………………………… ☐ 1 Inde ☐ 0 Ayi
5. Ulula zotsatila zanu kwa okondedwa anu………………… ☐ 1 Inde ☐ 0 Ayi
6. Ulula zotsatila zanu kwa abale anu………………………… ☐ 1 Inde ☐ 0 Ayi

Ngati otenga mbali anayamba ma ARV chifukwa cha thanzi lawo (pitani ku funso A pamwambapo) → Skip to #11

Ngati otenga mbali anayamba kumwa ma ARV ndi ndondomeko ya Option B+ (ali ndi mimba kapena akuyamwitsa) pitilizani ndi funso 7

7. Mabadwidwe a mwana wanu otsiriza mu ndondomeko ya Option B+ - singleton or 1st of multiple births:

Child #	1. ZOTSATIRA ZA HIV	2. UMOYO WAKE
a. 1 (singleton or 1st of multiple births)	☐ 0 Alibe HIV<br>☐ 1 Ali ndi HIV<br>☐ 2 Zotsatira za HIV sizikudziwika	☐ 0 Alimoyo<br>☐ 1 Anamwaliera asanakwene masiku 28<br>☐ 2 Anamwalira asanafike chaka chimodzi
b. 2 (2nd of multiple births)<br>☐ 1 Applicable ----------→<br>☐ 0 N/A → SKIP to #8	☐ 0 Alibe HIV<br>☐ 1 Ali ndi HIV<br>☐ 2 Zotsatira za HIV sizikudziwika	☐ 0 Alimoyo<br>☐ 1 Anamwaliera asanakwene masiku 28<br>☐ 2 Anamwalira asanafike chaka chimodzi

8. Momwe zinalili pa nkhani ya banja pa mimba yotsiriza mu ndondomeko ya Option B+:
   - ☐ 0 Osakwatiwa (sanakwatiwepo)
   - ☐ 1 Okwatiwa (akukhalira limodzi)
   - ☐ 2 Amakhala ndi chibwenzi koma sanakwatirane
   - ☐ 3 Anasiyana / okwatiwa koma amakhala kosiyana
   - ☐ 4 Banja linatha ndipo sakukhala ndi mwamuna wina aliyense
   - ☐ 5 Mwamuna wawo anamwalira ndipo sakukhala ndi mwamuna wina
9. Kodi mwamuna amene wakupatsani mimbayi ndi yemwenso amene munali naye pa mimba yotsiriza mu ndondomeko ya Option B+? ☐ 1 Inde ☐ 0 Ayi
10. Mwasinthapo malo okhala kuchokera pamene munakhala ndi mimba yotsiriza mu ndondomeko ya Option B+?
    ☐ 1 Inde ☐ 0 Ayi
11. a. Kodi ndi zifukwa ziti zimene munasiyira ndondomeko ya ART (*allow participant to answer freely before asking items 11b-v; record response here)*:____

____

***[Read to participant**: Tsopano ndikufunsani zufukwa zosiyanasiyana zimene munasiyira. Chonde ndiuzendi ngati zifukwazi zinakupangitsani "kwambiri", "pang'ono" kapena "palibe" kuti musiye mankhwala a HIV]*

**Kodi zifukwa izi zinathandiza bwanji kuti inuyo musiye?** *Funsani lililonse mwa mafunsowa b-v*	
11b. Basi sindinafune kumamwa mankhwala	☐ 2 Kwambiri ☐ 1 Pang'ono ☐ 0 Palibe
11c. Zikhulupiliro za chipembedzo/mpingo (anakhulupilira kuti anachilitsidwa)	☐ 2 Kwambiri ☐ 1 Pang'ono ☐ 0 Palibe

Staff Initials: Filled form_______ Staff initials: entered form into database: ____

S4 DEFAULTER QUESTIONNAIRE (DQ-1)

Patient ID ☐☐☐☐-☐☐-☐☐ Date of Patient Visit ☐☐ ☐☐☐ ☐☐☐☐ (D D M M M Y Y Y Y)

Step Number ☐ Visit code: ☐☐.☐

**Kodi zifukwa izi zinathandiza bwanji kuti inuyo musiye?** *Funsani lililonse mwa mafunsowa b-v*	
11d. Zikhulupiliro za chikhalidwe/Mizimu	☐$_2$ Kwambiri ☐$_1$ Pang'ono ☐$_0$ Palibe
11e. Kuopa zotsatira zoyipa za mankhwala	☐$_2$ Kwambiri ☐$_1$ Pang'ono ☐$_0$ Palibe
11f. Simunalandire chithandizo kuchoka kwa mamuna wanu (banja linatha/anasiyana)	☐$_2$ Kwambiri ☐$_1$ Pang'ono ☐$_0$ Palibe
11g. Simumadwala	☐$_2$ Kwambiri ☐$_1$ Pang'ono ☐$_0$ None
11h. Ndinakhulupilira njira zina zothandiza kuti mwana asatengere HIV	☐$_2$ Kwambiri ☐$_1$ Pang'ono ☐$_0$ Palibe
11hi: Ngati "kwambiri kapena "pang'ono", *tchulani*	☐$_1$ Mankhwala ena ☐$_2$ Mankhwala achikuda
11i. Kusalidwa	☐$_2$ Kwambiri ☐$_1$ Pang'ono ☐$_0$ Palibe
11ii: Ngati "kwambiri" kapena "pang'ono", *tchulani (check all that apply)*:	☐$_1$ Kusalidwa ndi apabanja<br>☐$_2$ Kusalidwa ndi achibale<br>☐$_3$ Kusalidwa ndi anthu a mdela langa<br>☐$_4$ Zina *(tchulani)* :*11ii4i*_______________
11j. Mutabereka mwana simunaonenso chifukwa chopitilizira mankhwala	☐$_2$ Kwambiri ☐$_1$ Pang'ono ☐$_0$ Palibe
11k. Mutasiya kuyamwitsa simunaonenso chifukwa chopitilizira mankhwala	☐$_2$ Kwambiri ☐$_1$ Pang'ono ☐$_0$ Palibe
11l. Amuna anu anapangitsa kuti musiye mankhwala	☐$_2$ Kwambiri ☐$_1$ Pang'ono ☐$_0$ Palibe
11m. Abale anu anapangitsa kuti musiye mankhwala	☐$_2$ Kwambiri ☐$_1$ Pang'ono ☐$_0$ Palibe
11n. Anthu okhala mdera lanu anapangitsa kuti musiye kumwa mankhwala	☐$_2$ Kwambiri ☐$_1$ Pang'ono ☐$_0$ Palibe
11ni. Ngati "kwambiri" kapena "pang'ono", *tchulani anthu ake*:	
11o. Munalibe nthawi yopitila ku chipatala kukalandira chithandizo cha mankhwala a HIV	☐$_2$ Kwambiri ☐$_1$ Pang'ono ☐$_0$ Palibe
11oi. Ngati "kwambiri " kapena "pang'ono , *tchulani*	☐$_1$ Ulimi<br>☐$_2$ Wa geni<br>☐$_3$ Ku malo antchito samalola<br>☐$_4$ Ndinachokapo<br>☐$_5$ Zina *(tchulani)* *11oi5i* ______________
11p. Chipatala cha ART chinali kutali	☐$_2$ Kwambiri ☐$_1$ Pang'ono ☐$_0$ Palibe
11q. Chithandizo chosakwanira pa chipatala cha ART	☐$_2$ Kwambiri ☐$_1$ Pang'ono ☐$_0$ Palibe
11qi. Ngati "kwambiri" kapena "pang'ono", *tchulani (check all that apply)*:	☐$_1$ Kusowa kwa mankhwala a ART<br>☐$_2$ Mankhwa kupezeka mwa apo ndi apo<br>☐$_3$ Kudikira nthawi yaitali<br>☐$_4$ Kunalibe ogwira ntchito kuchipatala<br>☐$_5$ Makhalidwe a anthu ogwira ntchito kuchipatala cha ART<br>☐$_6$ Zina (*tchulani*):11qi6i:_________________
11r. Ndimafuna chipatala cha private cha ART	☐$_2$ Kwambiri ☐$_1$ Pang'ono ☐$_0$ Palibe
11s. Zotsatira zoyipa za mankhwala a HIV	☐$_2$ Kwambiri ☐$_1$ Pang'ono ☐$_0$ Palibe
11si. Ngati "kwambiri" kapena "pang'ono", *tchulani (check all that apply)*:	☐$_1$ Zizindikiro zokhudza ubongo<br>☐$_2$ Zizindikiro zokhudza Mmimba<br>☐$_3$ Nsungu<br>☐$_4$ Chikasu<br>☐$_5$ Mavuto a mchikhodzodzo<br>☐$_6$ Other (*specify*): 11si6i:__________________
11t. Anakhulupilira kuti chitetezo chawo ndi chokwera	☐$_2$ Kwambiri ☐$_1$ Pang'ono ☐$_0$ Palibe

S4 DEFAULTER QUESTIONNAIRE (DQ-1)

Patient ID ☐☐☐☐-☐☐-☐☐ Date of Patient Visit ☐☐ ☐☐☐ ☐☐☐☐ (D D M M M Y Y Y Y)

Step Number ☐ Visit code: ☐☐.☐

**Kodi zifukwa izi zinathandiza bwanji kuti inuyo musiye?** *Funsani lililonse mwa mafunsowa b-v*	
11u. Zotsatira za mwana	☐ 2 Kwambiri ☐ 1 Pang'ono ☐ 0 Palibe
11ui. Ngati "kwambiri " kapena "pang'ono ", *tchulani*	
11v. Zina	☐ 2 Kwambiri ☐ 1 Pang'ono ☐ 0 Palibe
11vi. Ngati "kwambiri" kapena "pang'ono", *tchulani*	

12. a. Kodi zifukwa zimene mwalowelanso mu ndondomeko ya ya mankhwala a HIV a ART (*allow participant to answer freely before asking items 12b-k; record response here)*:______________________________________

____

*[**Read to participant**: ndikufunsani mafunso osiyanasiyana okhudza kulowanso kwanu mu ndondomeko yoyambanso* mankhwala a HIV a *ART. Chonde ndiuzeni ngati zifukwazi zinakupangitsani "kwambiri", "pang'ono" kapena "palibe" kuti mubwerenso mu ndondomeko imeneyi ya* ya mankhwala a HIV a *ART]*

**Kodi zifukwa izi zinathandiza bwanji kuti inuyo muyambirenso ndondomeko** ya mankhwala a HIV a **ART?** *Funsani lililonse mwa mafunsowa b-k*		
12b. Ndondomeko ya maphunzilo oonjezera a kusikelo ya amayi oyembekezera	☐ 2 Kwambiri ☐ 1 Pang'ono ☐ 0 Palibe	
12c. Chifukwa cha mimba ya ponoyi (mwangobwerera ndondomeko yoteteza mwana kuti asatengere kachilombo ka HIV pa mimba iyi)	☐ 2 Kwambiri ☐ 1 Pang'ono ☐ 0 Palibe	
12d. Mwaphunzitsidwanso za za mankhwala a HIV a ART koma osati pa sekelo ya yoyembekezera ya pano: 12di. Ngati "kwambiri" kapena "pang'ono", *tchulani*	☐ 2 Kwambiri ☐ 1 Pang'ono ☐ 0 Palibe<br>☐ 1 Zipatala zina Ma ARV<br>☐ 2 Malo ogona kuchipatala<br>☐ 3 O malo olembetsa matenda kuchipatala<br>☐ 4 Ma pologalamu ena a zaumoyo za mmadela	
12e.Matenda a mayi : 12ei: Ngati "kwambiri" kapena "pang'ono", *tchulani*	☐ 2 Kwambiri ☐ 1 Pang'ono ☐ 0 Palibe<br>Matenda:	
12f. Sindikudwala panopa (Poyamba ndinadwalika kwambiri, sindinakatha kubwera ku chipatala):	☐ 2 Kwambiri ☐ 1 Pang'ono ☐ 0 Palibe	
12g. Momwe zotsatira za mwana zinalili 12gi. & gii. Ngati "kwambiri " kapena "pang'ono," tchulani Zotsatira za HIV za mwana ndinso thanzi lake:	☐ 2 Kwambiri ☐ 1 Pang'ono ☐ 0 Palibe<br>ZOTSATIRA ZA HIV: 12gi.<br>☐ 0 Alibe HIV<br>☐ 1 Ali ndi HIV<br>☐ 2 Zotsatira za HIV sizikudziwika	UMOYO WAKE: 12gii.<br>☐ 0 Alimoyo<br>☐ 1 Anamwalira asanakwene masiku 28<br>☐ 2 Anamwalira asanafike chaka 1
12h. Kutsika kwa chitetezo	☐ 2 Kwambiri ☐ 1 Pang'ono ☐ 0 Palibe	
12i. Kupita patsogolo kwa chithandizo choteteza mwana kuti asatenge HIV 12ii. Ngati "kwambiri" kapena "pang'ono", *tchulani* *(check all that apply)*:	☐ 2 Kwambiri ☐ 1 Pang'ono ☐ 0 Palibe<br>☐ 1 Kupezeka kwa mankhwala kunapita patsogolo<br>☐ 2 Makhalidwe a anthu ogwira ntchito anasintha<br>☐ 3 Nthawi yodikira inachepa<br>☐ 4 Kuchuluka kwa anthu ogwira ntchito kuchipatala<br>☐ 5 Kupezeka kwa zipatala zotenegerako mankhwala a HIV *(tchulani)* 12ii5: ______________________<br>☐ 6 Zina *(tchulani):* 12ii6:______________________	
12j. Kutha kwa mchitidwe osalana 12ji. Ngati "kwambiri " kapena "pang'ono", *tchulani*:	☐ 2 Kwambiri ☐ 1 Pang'ono ☐ 0 Palibe	

Staff Initials: Filled form_______ Staff initials: entered form into database: ____

S4 DEFAULTER QUESTIONNAIRE (DQ-1)

Patient ID ☐☐☐☐-☐☐-☐☐ Date of Patient Visit ☐☐ ☐☐☐ ☐☐☐☐
D D M M M Y Y Y Y

Step Number ☐ Visit code: ☐☐.☐

**Kodi zifukwa izi zinathandiza bwanji kuti inuyo muyambirenso ndondomeko** ya mankhwala a HIV a **ART?** *Funsani lililonse mwa mafunsowa b-k*	
12k. Zifukwa zina	☐ 2 Kwambiri ☐ 1 Pang'ono ☐ 0 Palibe
12ki. Ngati "kwambiri" kapena "pang'ono" *tchulani*	

13a. Kodi otenga nawo mbali ayambanso kumwa ma ARV mu masiku asanu ndi awiri apitawa?

☐ 1 Inde ☐ 0 Ayi

13b. *Ngati ndi choncho:* Tsiku limene ayambiranso kumwa mankhwala: ☐☐ ☐☐☐ ☐☐ (*dd* *MMM* *yy*)

[END OF FORM]

Staff Initials: Filled form_______ Staff initials: entered form into database: ____

S4 ARV REGIMEN LOG (TX-1)

Patient ID ☐☐☐☐-☐☐-☐☐ Date of Patient Visit ☐☐ ☐☐☐ ☐☐☐☐ (D D MMM Y Y Y Y)

Page #:_____ Step Number ☐ Visit code: ☐☐.☐

Instructions:

COHORT A & C: *When starting a new or modified regimen*, ***complete Part A*** *(fill at first visit after starting regimen)*

COHORT B: *at enrollment step 1, fill parts A & B for all previous regimens. Part A only should be filled for the **current** regimen.*

ALL COHORTS: *When stopping or modifying the current regimen*, ***complete Part B*** *of this form and complete **Part A of a new Antiretroviral Regimen Log*** *with the new or modified regimen.*

PART A (starting new or modified regimen)

1. Regimen Start Date ☐☐ (dd) ☐☐☐ (MMM) ☐☐ (yy)

2. Visit code # at which regimen was started
☐☐.☐ OR: ☐ 999 N/A

3. This regimen is ☐ 1 initial ☐ 2 secondary ☐ 3 salvage

4. Malawi Guidelines Regimen (check one):
☐ 0 0A ☐ 2 2A ☐ 4 4A ☐ 5 5A ☐ 6 6A
☐ 7 7A ☐ 8 8A ☐ 9 9A ☐ 10 Other (specify):________

5. **REGIMEN MEDICATIONS:**
Codes: see back of page
Frequency: write "1" for once daily (od) OR "2" for twice daily (bd)

	**Med Code**	**Frequency**
5a.	☐☐	☐
5ai: specify if med code is "other": ______________		
5b	☐☐	☐
5bi: specify if med code is "other": ______________		
5c	☐☐	☐
5ci: specify if med code is "other": ______________		

5d. Clinic staff initials ☐☐☐

5e. Data entry staff initials ☐☐☐

PART B (stopping or modifying regimen from part A)

6. Regimen Stop/Modification Date: ☐☐ (dd) ☐☐☐ (MMM) ☐☐ (yy)

7. Visit code # at which regimen was stopped or modified
☐☐.☐ OR: ☐ 999 N/A

[proceed to #8]

8. **Medication Status** — **i. Stop/Mod Codes** **(see back of this page for codes)**

	No change	Dose/freq. change	stopped	Held	primary	secondary
5a	☐	☐	☐	☐	☐☐☐	☐☐☐
5b	☐	☐	☐	☐	☐☐☐	☐☐☐
5c	☐	☐	☐	☐	☐☐☐	☐☐☐

9. Were any of the above stop/modification codes reported as AE? ☐ 1 Yes ☐ 0 No
→ Record AE Log page(s):
9i: __ __ 9ii: __ __
9iii: __ __ 9iv: __ __

10. Clinic staff initials ☐☐☐

10a. Data entry staff initials ☐☐☐

S4 ARV REGIMEN LOG (TX-1)

[see codes for questions #4, 5, and 8 on the back of this page]

S4 ARV REGIMEN APPENDIX

Codes for questions #4, 5, and 8

For Question #4		For Question #5
**Malawi ART Regimens**	**Maternal Study Drug Formulation**	**CODE COMBOS**
5A	TDF300mg/3TC300mg/EFV600mg	**10**
2A	AZT300mg/3TC150mg/NVP200mg	**11**
6A	TDF300mg/3TC300mg + NVP200mg	**13 + 16**
4A	AZT300mg/3TC150mg + EFV600mg	**12 + 15**
0A	ABC600mg/3TC300mg + NVP200mg OR: ABC 300mg/3TC150mg + NVP 200mg	**14 + 16**
7A	TDF300mg/3TC300mg + ATV300mg/r100mg	**13 + 17**
8A	AZT300mg/3TC 150mg + ATV300/r100mg	**12 + 17**
9A	ABC600mg/3TC300mg + ATV 300/r100mg	**14 + 17**
1A	d4T 30mg/3TC 150mg/NVP 200mg	**20**
3A	d4T 30mg/3TC 150mg + EFV 600mg	**21 + 15**

Medication regimen CODES (numeric order)	
**10**	TDF/3TC/EFV
**11**	AZT/3TC/NVP
**12**	AZT/3TC
**13**	TDF/3TC
**14**	ABC/3TC
**15**	EFV
**16**	NVP
**17**	ATV/r
**18**	LPV/r
**19**	Other (specify)
**20**	d4T/3TC/NVP
**21**	d4T/3TC

Stop/Modification Code List (for #8i)

115 Start of subsequent step (use for cohort C step 2)
199 Completed protocol defined period of evaluation/treatment (to close infant NVP at week 6 and close drugs at study exit)

Clinical Toxicities
205 Anemia
398 Combination clinical toxicities, specify
399 Clinical toxicity, other, not listed in Appendix 71, specify

Laboratory Toxicities
400 Absolute neutrophil count (ANC), decreased
405 ALT (SGPT), increased
407 AST (SGOT), increased
424 Creatinine clearance, calculated, decreased
438 Hemoglobin, increased
439 Hemoglobin, decreased
446 Lymphocyte count, increased
447 Lymphocyte count, decreased
450 Neutropenia
454 Platelets, decreased
455 Platelets, increased
468 WBCs, increased
469 WBCs, decreased
472 ANC, increased
482 Hematologic laboratory toxicity, specify toxicity(ies)
483 Chemistry laboratory toxicity, specify toxicity(ies)
598 Combination laboratory toxicities, specify
599 Laboratory toxicity, other, not listed in Appendix 71, specify

Clinical Events or Progression
600 AIDS-defining illness
685 Infant HIV positive (use for mothers only)
687 Confirmed HIV infection (Use for infant only)

Obstetrical Complications (Use for maternal participants)
702 Fetal demise
710 Eclampsia

Immunologic Failure
803 Protocol defined immunologic decline

Virologic Failure
852 Protocol defined virologic failure

Toxicity Decreased/Resolved
970 Toxicity decreased/resolved, specify toxicity

Clinician/Study Participant Request/Decision
981 Clinician request/decision, specify reason
982 Study Participant/Guardian request/decision, specify reason

Non-compliance (for Permanent D/C only)
983 Non-compliant with study visits, specify reason
984 Non-compliant with study medications, specify reason
985 Non-compliant with study visits and study medications, specify reason
989 Protocol step change other than protocol-designed interruption

Other
997 Death
998 Combination of reasons, specify
999 Other, not listed in Appendix 71, specify

S4 HIV TESTING & TREATMENT HISTORY RECORD (HVT-2)

Patient ID ☐☐☐☐-☐☐-☐☐ Date of Patient Visit ☐☐ ☐☐☐ ☐☐☐☐ (D D MMM Y Y Y Y)

Step Number ☐ Visit code: ☐☐.☐

1. Is this your first pregnancy? .... ☐$_1$ Yes (→ SKIP to #4a) ☐$_0$ No
2. Have you been pregnant since 2011? .... ☐$_1$ Yes ☐$_0$ No (→ SKIP to #4a)
3. Were you tested for HIV with that (previous) pregnancy? ☐$_1$ Yes (→ SKIP to #4b) ☐$_0$ No

3a. If NO, why did you **not** get an HIV test in that previous pregnancy?

☐$_1$ No tests at facility ☐$_4$ Test not offered
☐$_2$ No counselor at facility ☐$_5$ Other (*specify*) 3ai:________________________________
☐$_3$ Did not go to ANC

4.a. You tested for HIV this week. Was this your first HIV test?

☐$_1$ Yes → (SKIP to Question #5) ☐$_0$ No ☐$_2$ Don't know ☐$_3$ No response

4b. What was the date of your last HIV test? ☐☐ (dd) ☐☐☐ (MMM) ☐☐ (yy) 4bi☐$_9$ Estimated (not exact date)

4c. What setting were you tested in? ☐$_1$ ANC ☐$_2$ Under 5 ☐$_3$ HTC/ART clinic ☐$_4$ TB ☐$_5$ Other

4d. What was the result of your last test? ☐$_1$ Positive ☐$_0$ Negative ☐$_2$ Indeterminate ☐$_4$ Don't know
(Negative, Indeterminate) → SKIP to #6

4e. Did you receive a CD4 count at that time? ☐$_1$ Yes ☐$_0$ No ☐$_2$ Don't know ☐$_3$ No response
(No, Don't know, No response) → SKIP to #4g

4f. CD4 value: __ __ __ OR: ☐$_0$ Not available

4g. Were you offered ART at that time? ☐$_1$ Yes ☐$_0$ No ☐$_2$ Don't know ☐$_3$ No response

4h. Did you start taking ART at that time? ☐$_1$ Yes ☐$_0$ No ☐$_2$ Don't know ☐$_3$ No response

4hi. If NO to 4h, what was the primary reason why not? (*Tick only ONE "primary reason" box)*

Were any of these other items reasons why you did not start ART at that time?
(***ASK ABOUT EACH ITEM,*** *check "yes" or "no" to each non-primary reason*)

1. Simply did not want to take drugs	☐$_1$ Yes ☐$_0$ No ☐$_2$ PRIMARY REASON
2. Religious reasons	☐$_1$ Yes ☐$_0$ No ☐$_2$ PRIMARY REASON
3. Traditional/spiritual beliefs	☐$_1$ Yes ☐$_0$ No ☐$_2$ PRIMARY REASON
4. Fear of side effects	☐$_1$ Yes ☐$_0$ No ☐$_2$ PRIMARY REASON
5. Had no support of partner (separated/divorced/relationship problem)	☐$_1$ Yes ☐$_0$ No ☐$_2$ PRIMARY REASON
6. Was not feeling ill	☐$_1$ Yes ☐$_0$ No ☐$_2$ PRIMARY REASON
7. ART clinic too far	☐$_1$ Yes ☐$_0$ No ☐$_2$ PRIMARY REASON
8. Poor service at ART clinic	☐$_1$ Yes ☐$_0$ No ☐$_2$ PRIMARY REASON
9. Fear of stigma/discrimination	☐$_1$ Yes ☐$_0$ No ☐$_2$ PRIMARY REASON
10. Other (*specify*)4hio _______________	☐$_1$ Yes ☐$_0$ No ☐$_2$ PRIMARY REASON

****SKIP to #5 if 4hi is answered****

4i. Did you stop taking ART? ☐$_1$ Yes ☐$_0$ No ☐$_2$ Don't know ☐$_3$ No response
(No, Don't know, No response) → SKIP to #5

4j. When did you stop? (dd/mmm/yy) __ __/__ __ __/__ __

4k. Why did you stop? (*check ONE primary reason,* then ***ASK ABOUT EACH of 8 ITEMS:*** *check "yes" or "no" to each non-primary reason*)

1. Side effects	☐$_1$ Yes ☐$_0$ No ☐$_2$ PRIMARY REASON
2. Travel	☐$_1$ Yes ☐$_0$ No ☐$_2$ PRIMARY REASON
3. Pregnancy ended	☐$_1$ Yes ☐$_0$ No ☐$_2$ PRIMARY REASON
4. Breastfeeding ended	☐$_1$ Yes ☐$_0$ No ☐$_2$ PRIMARY REASON

Staff Initials: Filled form_______ Staff Initials: Entered form into Database_______

Patient ID ☐☐☐☐-☐☐-☐☐ Date of Patient Visit ☐☐ ☐☐☐ ☐☐☐☐ (D D M M M Y Y Y Y)

Step Number ☐ Visit code: ☐☐.☐

5. Felt healthy	☐1 Yes ☐0 No ☐2 PRIMARY REASON
6. ART clinic too far	☐1 Yes ☐0 No ☐2 PRIMARY REASON
7. Poor service at ART clinic	☐1 Yes ☐0 No ☐2 PRIMARY REASON
8. Other (*specify*)4ki ________________	☐1 Yes ☐0 No ☐2 PRIMARY REASON

5. Are you currently on ART? ☐1 Yes ☐0 No
   5a. If yes, why did you initiate ART?
   - ☐1 Current pregnancy
   - ☐2 Previous pregnancy – before delivery
   - ☐3 Previous pregnancy—after delivery while breastfeeding
   - ☐4 CD4 count below threshold
   - ☐5 Other (specify) 5ai: ____

6. Is this/will this be your first time to start ART because of pregnancy? ☐1 Yes ☐0 No

7. Have you ever had an HIV RNA test done? ☐1 Yes ☐0 No→(SKIP TO #8)
   If YES: 7a. HIV RNA result:________ (copies/mL) OR: ☐0 Not available
   7b. Date of test: (dd/mmm/yy) __ __/__ __ __/__ __

8. Ever taken ART? ☐1 Yes → *fill in table below with MOST RECENT drug(s)* ☐0 No→ SKIP to #13

Indication	Yes/No	Regimen	Start date	Stop Date or Ongoing?
**a. Own Health**	☐1 Yes→ a1<br>☐0 No→ SKIP to #8b	**a1.**	**a1i** ☐☐ ☐☐☐ ☐☐ (*dd MMM yy*)<br>OR: ☐99 Unavailable	**a1ii** ☐☐ ☐☐☐ ☐☐ (*dd MMM yy*)<br>**OR:** ☐98 **ongoing** ☐99 Unavailable
		**a2.**<br>☐99 N/A	**a2i** ☐☐ ☐☐☐ ☐☐ (*dd MMM yy*)<br>OR: ☐99 Unavailable	**a2ii** ☐☐ ☐☐☐ ☐☐ (*dd MMM yy*)<br>**OR:** ☐98 **ongoing** ☐99 Unavailable
**b. PMTCT**	☐1 Yes→ b1<br>☐0 No→ SKIP to #9	**b1.**	**b1i** ☐☐ ☐☐☐ ☐☐ (*dd MMM yy*)<br>OR: ☐99 Unavailable	**b1ii** ☐☐ ☐☐☐ ☐☐ (*dd MMM yy*)<br>**OR:** ☐98 **ongoing** ☐99 Unavailable
		**b2.**<br>☐99 N/A	**b2i** ☐☐ ☐☐☐ ☐☐ (*dd MMM yy*)<br>OR: ☐99 Unavailable	**b2ii** ☐☐ ☐☐☐ ☐☐ (*dd MMM yy*)<br>**OR:** ☐98 **ongoing** ☐99 Unavailable

9. Is there evidence of attendance to ART pick-up visits **in the last 3 months** (in mastercard or health passport)?
   ☐1 Yes ☐0 No ☐9 Not available ☐8 Not Applicable (only had non-HAART PMTCT)→ SKIP to #13

10. Are pick-up dates within 2 days of expected visit date (in mastercard or health passport)?
    ☐1 Yes ☐0 No ☐9 Not available

11. Do pill counts suggest 95% adherence or greater **in the past 3 months**? ☐1 Yes ☐0 No ☐9 Not available

12. Ask client, How many doses have you missed **in the last month**?
    ☐0 None ☐1 1 dose ☐2 2 doses ☐4 ≥3 doses

13. Are you currently taking any other treatments (including traditional medications)? ☐1 Yes ☐0 No
    If YES, *specify (13i)*:____
    ____ ***--> Complete Concomitant Medications Log***

14. Allergies: ☐1 Yes ☐0 No If YES, *specify* (*14i*):____

[END OF FORM]

Staff Initials: Filled form_______ Staff Initials: Entered form into Database_______
