# Supplementary material for: Factors associated with a history of treatment interruption among pregnant women living with HIV in Malawi: A cross-sectional study
Source: PLoS One. 2022 Apr 19;17(4):e0267085. doi: 10.1371/journal.pone.0267085 (PMC9017884; doi:10.1371/journal.pone.0267085)
Supplement: Attachment — Submitted filename: Response to Reviewers.docx [file pone.0267085.s002.docx]

**Response to Reviewers**

**Editor Comments**

1. The PLOS ONE style templates were reviewed and appropriate changes were made.

2. English and Chichewa questionnaires are uploaded to Supporting Information. Included are the maternal enrollment form in English and Chichewa, the reinitiator (‘Defaulter’) questionnaire in English and Chichewa, the ARV regimen log in English, and the HIV testing and treatment form in English.

3. To allow comparison with other local data sources, the questionnaires included commonly used fields in the Malawi Demographic and Health Survey, or were adapted from case report forms of previous clinical trials conducted at the same site in Lilongwe, Malawi.

4. We have ensured that the grant information provided in the Funding Information and Financial Disclosure sections match.

5. The minimum necessary data are provided on the figshare repository.

**Reviewer #1**

1. Thank you for your suggestion. We agree that clarification of the study population was warranted and changed the language throughout the manuscript to reflect that we studied women reinitiating care as opposed to women truly lost to follow up. We have made this change in the title: “Factors associated with a history of treatment interruption among pregnant women living with HIV in Malawi: A cross sectional study,” the abstract (ex/ line 40, 43), and the main text (ex/ line 102).

2. We thank the reviewer for the recommendation of a more nuanced discussion of free response reasons for ART discontinuation. We agree that given the interview setting within an antenatal clinic, respondents could be more likely to report external factors beyond their control. We have included this discussion in lines 423-431, and included the reference suggested:

Zhou A. The uncertainty of treatment: Women’s use of HIV treatment as prevention in Malawi. Soc Sci Med. 2016;158:52–60.

3. “Documentation of HIV-positive status while pregnant or breastfeeding after July 2011 (when the implementation of Option B+ began)” was part of inclusion criteria to capture women likely offered ART but unwilling to disclose that they had interrupted therapy due to high uptake of ART. However, no women in the study fit this criterion.

**Reviewer #2**

1,2. Thank you for your suggestion - three senior researchers included in the co-authors have reviewed the paper for readability.

3. We thank the reviewer for raising these important points and believe we have chosen the most appropriate statistical methods. Our primary outcome of interest is a binary outcome (LTFU). When a binary outcome is being estimated, either a logistic regression model (to estimate an odds ratio) or a log-binomial regression model (to estimate a prevalence ratio) is typically used. However, sometimes, a log-binomial regression model does not converge. In these instances, a modified Poisson regression model can be used to approximate a prevalence ratio. We prefer a prevalence ratio to an odds ratio due to ease of interpretation. We provide the following citations explaining the mathematical underpinnings in greater detail:

14.	Zou G. A Modified Poisson Regression Approach to Prospective Studies with Binary Data. Am J Epidemiol. 2004;159(7):702–6.

15.	Barros A, Hirakata V. Alternatives for logistic regression in cross-sectional studies: an empirical comparison of models that directly estimate the prevalence ratio. BMC Med Res Methodol. 2003;3(21):16–21.

We have added the word “modified” in the abstract and text for greater clarity (line 48, 170, 172). In the text we explain this decision in the following way:

“To explore potential factors associated with LTFU, we used modified Poisson regression models with robust variance to calculate unadjusted prevalence ratios (PR). Modified Poisson regression with robust variance estimates were utilized due to issues with convergence of log-binomial regression models (14).”

4. We have clarified our process of quantification of qualitative responses in the methods section in lines 181-193, as also suggested by Reviewer #3. The description now reads as, “Qualitative free responses for ART discontinuation in the reinitiating cohort were organized using thematic content analysis (16). One researcher reviewed all participant responses to the question and developed eight categories for coding. Two independent researchers applied the codebook to the responses. The initial inter-rater reliability Cohen’s kappa was 0.86. Discrepancies were reviewed and resolved by consensus. The frequency of reason for ART discontinuation category was tabulated and reported.”

5. We were unable to substantially modify the length of the discussion due to suggestions by other reviewers. However, we take note the impression of Reviewer 3 who felt the manuscript was concise.

**Reviewer #3**

Thank you for your comments helpful for improved contextualization of our study. All of the patients presented at free will to an antenatal clinic and were subsequently approached (no tracing occurred)– we have provided clarification in lines 113-115 with the following description, “For this study, women who presented for an antenatal clinic visit and tested positive for HIV during any trimester of pregnancy were approached for recruitment into either: 1) the retained cohort, or 2) the reinitiating cohort.” To better address how the study results may have been influenced by the setting of the study, we have included lines 423-431 as described for Reviewer 1’s second point above. We have additionally clarified that qualitative responses were organized using a thematic approach and the frequencies were tabulated, as described in our response to Reviewer 2’s point 4. We unfortunately did not collect information on the prior location of ART provision in the reinitiating cohort, so we cannot provide this additional contextualization. The two sites, Area 18 Health center and Bwaila Hospital, are comparable in providing comprehensive antenatal and HIV care for patients. However, Area 18 is smaller and less busy.
